# Non-coding variants in *VAMP2* and *SNAP25* affect gene expression: potential implications in migraine susceptibility

**DOI:** 10.1186/s10194-023-01615-z

**Published:** 2023-06-29

**Authors:** Daniela Felício, Andreia Dias, Sandra Martins, Estefânia Carvalho, Alexandra M. Lopes, Nádia Pinto, Carolina Lemos, Mariana Santos, Miguel Alves-Ferreira

**Affiliations:** 1 Instituto de Investigação e Inovação em Saúde (i3S), 4200-135 Porto, Portugal; 2 Institute of Molecular Pathology and Immunology of the University of Porto (IPATIMUP), 4200-135 Porto, Portugal; 3 ICBAS - School of Medicine and Biomedical Sciences, Universidade Do Porto, 4050-313 Porto, Portugal; 4 Unit for Genetic and Epidemiological Research in Neurological Diseases (UnIGENe), Instituto de Biologia Molecular e Celular (IBMC), Universidade do Porto, 4200-135 Porto, Portugal; 5 Centre for Predictive and Preventive Genetics (CGPP), Instituto de Biologia Molecular e Celular (IBMC), Universidade do Porto, 4200-135 Porto, Portugal; 6 Centro de Matemática da Universidade do Porto (CMUP), 4169-007 Porto, Portugal

**Keywords:** Migraine, Reporter gene assays, SNARE complex, Non-coding variants, *VAMP2*, *SNAP25*, *STX1A*, Gene expression

## Abstract

Migraine is a common and complex neurological disease potentially caused by a polygenic interaction of multiple gene variants. Many genes associated with migraine are involved in pathways controlling the synaptic function and neurotransmitters release. However, the molecular mechanisms underpinning migraine need to be further explored.

Recent studies raised the possibility that migraine may arise from the effect of regulatory non-coding variants. In this study, we explored the effect of candidate non-coding variants potentially associated with migraine and predicted to lie within regulatory elements: *VAMP2*_rs1150, *SNAP25*_rs2327264, and *STX1A*_rs6951030. The involvement of these genes, which are constituents of the SNARE complex involved in membrane fusion and neurotransmitter release, underscores their significance in migraine pathogenesis. Our reporter gene assays confirmed the impact of at least two of these non-coding variants. *VAMP2* and *SNAP25* risk alleles were associated with a decrease and increase in gene expression, respectively, while *STX1A* risk allele showed a tendency to reduce luciferase activity in neuronal-like cells. Therefore, the *VAMP2*_rs1150 and *SNAP25*_rs2327264 non-coding variants affect gene expression, which may have implications in migraine susceptibility. Based on previous in silico analysis, it is plausible that these variants influence the binding of regulators, such as transcription factors and micro-RNAs. Still, further studies exploring these mechanisms would be important to shed light on the association between SNAREs dysregulation and migraine susceptibility.

## Background

Migraine is a common disabling multifactorial neurological disease with a heritability estimated between 30–60% [1, 2]. Migraine affects about 15% of the population and is three times more prevalent in women [2]. This type of primary headache typically causes recurrent attacks of unilateral throbbing pain along with other symptoms, such as photophobia, nausea, and/or vomiting [3]. There are two common migraine subtypes defined by the presence or absence of aura [3]. Rare monogenic forms of familial hemiplegic migraine are caused by variants in genes related to neurotransmission (*CACNA1A*, *ATP1A2*, and *SCN1A*) [1]. However, many migraine cases remain without a genetic cause probably because common forms of migraine result from the contribution of multiple variants with small effects at several loci [4–6]. Most of the genes associated with migraine are involved in the metabolism, transport, and reception of neurotransmitters, possibly causing an imbalance among them, and consequently altering the synaptic function [7].

Studies indicate that migraine possibly results from an altered state of neuronal excitability driven by enhanced responsiveness to stimuli or abnormal processing of sensory information [1, 8]. Regulation of the expression of genes involved in the release of neuropeptides/neurotransmitters may have implications in migraine susceptibility [9–11]. Additionally, neurovascular mechanisms may underlie migraine pathophysiology, as shown by a recent genome-wide study, in which risk variants were enriched in both vascular and central nervous system tissues [12, 13].

Following the first hypothesis, our group explored the association of variants in genes belonging to the synaptic vesicle machinery and neurotransmission pathway through gene candidate association studies [14–16]. From the candidate variants identified in these studies, we have previously performed an in silico analysis of non-coding variants using scoring methods and epigenetic databases, which resulted in the selection of three variants within regulatory elements: *VAMP2*_rs1150 (3′ UTR), predicted as a target of a miRNA; *SNAP25*_rs2327264, (distal enhancer), expected to lie within a binding site of a transcription factor; and *STX1A*_rs6951030 (proximal enhancer), predicted to affect the binding affinity of zinc-finger transcription factors and disturb *TBL2* gene expression [17]. To note that *VAMP2*, *SNAP25* and *STX1A* genes encode presynaptic proteins that belong to the SNARE complex (soluble *N*-ethylamine-sensitive factor attachment protein receptor), which is involved in plasma membrane fusion and neurotransmitter release during synaptic transmission [18]. From these non-coding variants, at least *VAMP2*_rs1150 was previously associated with attention deficit hyperactivity disorder (ADHD) and working memory in addition to migraine susceptibility [16, 19].

In this study, we explored for the first time the effect of these three non-coding variants on gene expression, which may have implications in migraine susceptibility or other complex diseases related to SNARE dysfunction.

## Methods

### Cell culture

HEK293T cells (ATCC) were cultured in high glucose in Dulbecco’s modified Eagle medium (DMEM, GlutaMAX™) supplemented with 10% fetal bovine serum (FBS) and 1% antibiotic/antimycotic (Gibco, ThermoFisher Scientific, Waltham, MA, USA). SH-SY5Y cell line (DSMZ) was grown in DMEM GlutaMAX™/Ham’s F-12 nutrient mixture supplemented with 10% FBS and 1% antibiotic/antimycotic (Gibco, ThermoFisher Scientific, Waltham, MA, USA). HEK293T and SH-SY5Y cells were maintained at 37 °C in a humidified 5% CO2 atmosphere.

### Plasmids cloning

The plasmids were obtained by cloning the genomic sequences (length ~ 1500 bp) flanking the variants *VAMP2_*rs1150 (c.*1590 T > C) and *SNAP25_*rs2327264 (c.-64 + 6629 T > C) into the pGL3-promoter vector (Promega, Fitchburg, WI, USA). *VAMP2* exon 5 (3' UTR) and *SNAP25* intron 1 (enhancer) regions were PCR amplified from genomic DNA (Table 1), and PCR products were purified with Zymoclean Gel DNA Recovery Kit (Zymo Research, Irvine, CA, USA) and genotyped by Sanger sequencing. PCR products were inserted into the pGL3-promotor vector downstream of the firefly luciferase gene by Gibson Assembly (New England Biolabs, Ipswich, MA, USA) (Table 1).

**Table 1:** Primer sequences used for plasmids’ cloning

SNP	Lenght (bp)	Forward primer (5′-3′)	Reverse primer (5′-3′)
*VAMP2*_rs1150	1437	CTGAACGATGGctgaaatctctggcctcacc	GTTGAAGGCTCTCgttcaagcaattctctgcct
pGL3-promotor/*VAMP2*_rs1150	5028	gaattgcttgaacGAGAGCCTTCAACCCAGTC	cagagatttcagCCATCGTTCAGATCCTTATCG
*SNAP25*_rs2327264	1538	GATCTGAACGATGGgcagttccctcactcatc	GTTGAAGGCTCTCgaatgccataatagcagctg
pGL3-promotor/*SNAP25*_rs2327264	5028	ctattatggcattcGAGAGCCTTCAACCCAGTC	gagggaactgcCCATCGTTCAGATCCTTATCG

The portions regarding pGL3-promotor homologous ends are in uppercase letters

The genomic sequence (length of ~ 1500 bp) flanking *STX1A*_rs6951030 (c.30 + 691A > C) was obtained through the NZYTech Gene Synthesis service (NZYTech, Lisbon, Portugal). *STX1A* intron 1 (promotor) was cloned into the pGL3-basic (Promega, Fitchburg, WI, USA) upstream of the firefly luciferase gene by restriction with Nhel/Xhol (ThermoFisher Scientific, Waltham, MA, USA) enzymes.

Sequences were modified by site-directed mutagenesis to generate the alternative alleles (normal or risk allele) using the Q5 Site-directed mutagenesis kit (New England Biolabs, Ipswich, MA, USA), according to the manufacturer’s protocol. The following primer pairs were used to introduce c.*1590C > T (*VAMP2_*rs1150 normal allele), c.-64 + 6629 T > C (*SNAP25_*rs2327264 risk allele), and c.30 + 691A > C (*STX1A*_rs6951030 risk allele) variants: forward primer 5′-GTGCTGTGTTtTAGACCCCCC-3′ and reverse primer 5′-CCCCACCTCCAGCATCTC-3′; forward primer 5′-ATATGGTTCAcATTACTCAAAGATG-3′ and reverse primer 5′-CAACAACAGCAAAGAAGAG-3′; and forward primer 5′-TTCGGGCAGCcCTGGCTGGCG-3′ and reverse primer 5′-AGCCCGAAGGTGGATAGGTG-3′, respectively. All constructs were verified by Sanger sequencing.

### Cell transfection and dual-luciferase reporter gene assays

HEK293T and SH-SY5Y cells were transiently transfected for 48 h with pGL3-promotor-*SNAP25*, pGL3-promotor-*VAMP2*, pGL3-basic-*STX1A*, pGL3-control, pGL3-promoter, or pGL3-basic plasmids (150 ng; 96-well plate) (Promega, Fitchburg, WI, USA) using DreamFect Gold (OZ Biosciences, Marseille, Provence-Alpes-Cote d'Azur, France), according to the manufacturer’s protocol. Co-transfection with the pRL-CMV *renilla* vector (15 ng; 96-well plate) (Promega, Fitchburg, WI, USA) was used as an internal control for transfection efficiency in a 10:1 molar ratio (firefly:*renilla*). Dual-luciferase assays were performed in 96-well white plates (CELLSTAR® plates—µClear® bottom; Greiner Bio-One, Kremsmünster, Austria) containing 100 µL medium (without 1% antibiotic/antimycotic) with 1.5 × 10^4^ HEK293T cells/mL or 2.5 × 10^4^ SH-SY5Y cells/mL. After 48 h post-transfection, Synergy Mx Microplate Reader (Agilent, Santa Clara, CA, USA) was used to measure the luciferase activity with the Dual-Luciferase Reporter System (Promega, Fitchburg, WI, USA), according to the instructions recommended by the manufacturer.

### Statistical analysis

Statistical significance of the difference in the luciferase activity between normal and risk alleles was determined using unpaired student´s t-test; the threshold of statistical significance was set at *p* < 0.05. Statistical analysis was performed using the IBM SPSS Statistics 26.0 software (IBM, Armonk, NY, USA). Data was expressed as mean ± standard deviation (SD) considering at least four independent experiments and five replicates per experiment.

## Results

Recently, variants in the SNARE genes *VAMP2*, *SNAP25* and *STX1A* have been studied as potential risk factors in several neurological disorders, including migraine [15, 16, 20, 21]. Thus, following our previous in silico analysis, in which the non-coding variants *VAMP2*_rs1150 (3’ UTR), *SNAP25*_rs2327264 (distal enhancer), and *STX1A*_rs6951030 (proximal enhancer) were predicted to have high regulatory potential, we decided to confirm the effect of these candidate variants on gene expression through reporter gene assays [17]. After cloning the DNA sequences surrounding the variants, plasmids were transfected into two cell lines, one non-neuronal (HEK293T) and one neuronal-like (SH-SY5Y), and the luciferase gene reporter activity measured by a luminescence assay. The luciferase activity in transfected cells is approximately proportional to the mRNA levels, being used as a tool to study gene expression at the transcriptional level [22].

**Fig. 1:**
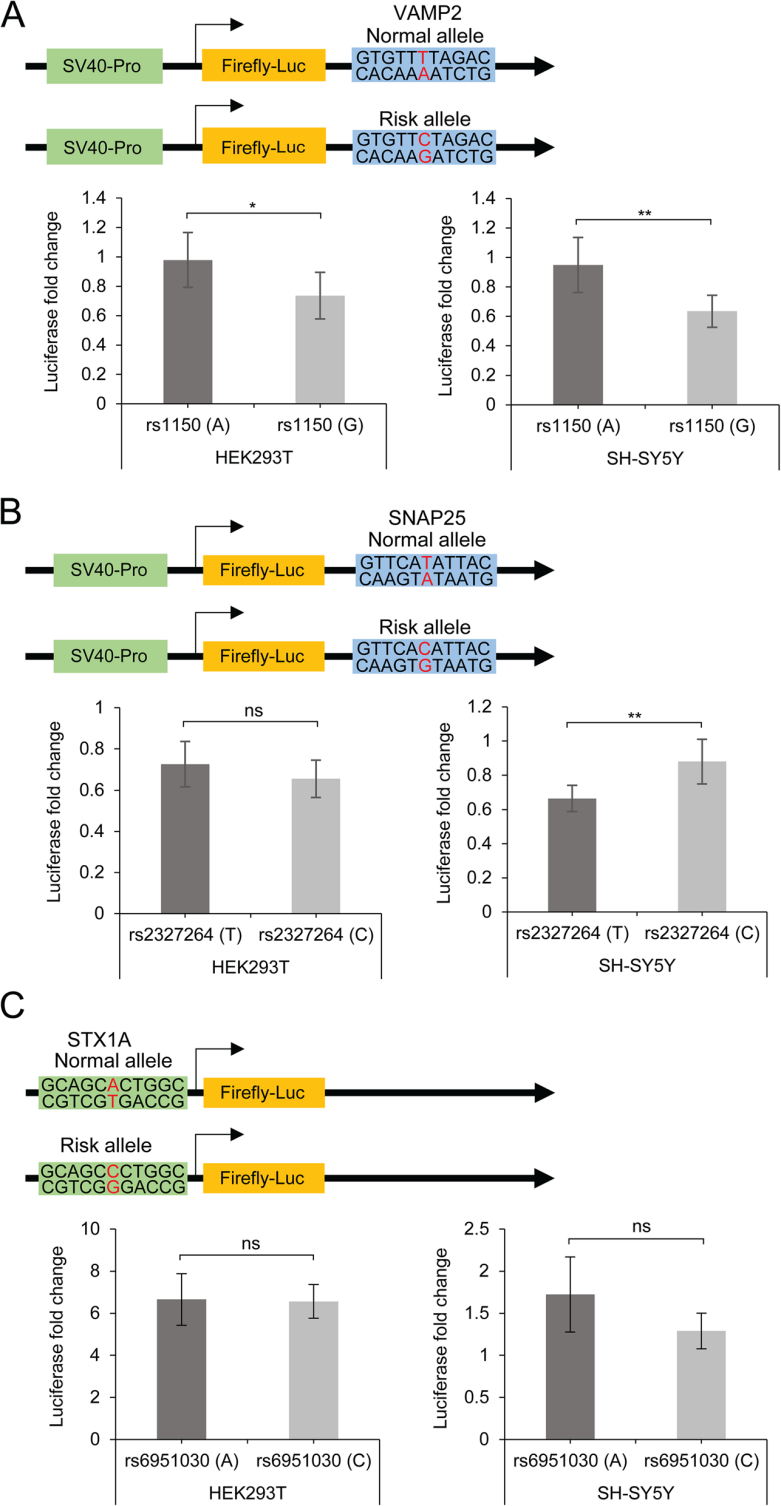
Reporter gene assays showed that allelic differences at *VAMP2*_rs1150 (**A**) and *SNAP25*_rs2327264 (**B**), but not at *STX1A*_rs6951030 (**C**), influenced luciferase reporter activity. Firefly luciferase activity was normalised to *renilla* luciferase activity and is shown as a fold change to that of pGL3-promotor or pGL3-basic (*n* ≥ 4 for each group) for HEK293T and SH-SY5Y cells. Data is presented as the mean ± SD. ns, not significant, * *p* < 0.05, ** *p* < 0.01, unpaired student´s t-test

We compared the luciferase activity driven by the different alleles: *VAMP2*_rs1150 G-allele (risk allele) *versus* A-allele (normal allele), *SNAP25*_rs2327264 C-allele (risk allele) *versus* T-allele (normal allele), and *STX1A*_rs6951030 C-allele (risk allele) *versus* A-allele (normal allele). We found that *VAMP2*_rs1150 G-allele significantly decreased luciferase activity by 24% and 31% compared to the A-allele in HEK293T and SH-SY5Y cells (Fig. 1A, *p* = 0.022 and *p* = 0.005, respectively), respectively. On the other hand, *SNAP25*_rs2327264 C-allele significantly increased luciferase activity by ~ 20% compared to the T-allele only in SH-SY5Y cells (Fig. 1B, *p* = 0.006). There were no significant differences between *SNAP25*_rs2327264 alleles in HEK293T cells (Fig. 1B, *p* = 0.2999). Therefore, risk alleles in *VAMP2* and *SNAP25* seemed to have opposite effects on the regulation of gene expression in neuronal-like cells. *STX1A*_rs6951030 C-allele showed a tendency to reduce luciferase activity (~ 40%) in SH-SY5Y cells, when compared with the A-allele, but did not reach statistical significance in either cell line (Fig. 1C, *p* = 0.900 and *p* = 0.335, respectively).

## Discussion

In this study, we demonstrated that the potential regulatory variants *VAMP2*_rs1150 and *SNAP25*_rs2723264 have indeed an impact on gene expression. *VAMP2*_rs1150 G-allele (risk allele) significantly decreased luciferase activity, while *SNAP25*_rs2723264 C-allele (risk allele) increased luciferase activity when compared to the normal alleles in SH-SY5Y cells. Luciferase activity was not significantly affected by *SNAP25*_rs2723264 in HEK293T cells, probably because gene regulation is tissue and cell-specific. According to the Protein Atlas (https://www.proteinatlas.org/; accessed 03 January 2023), *SNAP25* expression is 46.6 and 0.3 normalized transcript per million (nTPM) in SH-SY5Y and HEK293T cells, respectively. Thus, it is likely that regulators targeting this enhancer are poorly expressed in HEK293T cells, explaining the lack of differences in the luciferase activity between alleles in this cell line. On the other hand, the expression of *VAMP2* and possibly of its gene regulators is more uniform and broader between cell types (37.8 and 36 nTPM in SH-SY5Y and HEK293T cells, respectively). Amongst the three genes, *STX1A* is the one with the lowest expression in these cell lines (22.5 and 3.9 nTPM in SH-SY5Y and HEK293T cells, respectively), which may explain the lack of statistical significance in our assays. Nevertheless, the results of the reporter gene assays provide evidence to support the effect of at least two non-coding variants here analysed. In addition, it would be interesting to explore the synergistic effect between these common variants and other variants located within the same regulatory elements.

Interestingly, our functional data partially support our previous in silico analysis [17]. *VAMP2*_rs1150 was our top candidate variant, with 7 scoring methods indicating deleteriousness, while *SNAP25*_rs2723264 and *STX1A*_rs6951030 were predicted to have similar potential to be deleterious (3 scoring methods, differing by a few decimals in the sum parameter) [17].

A previous study from our group has shown a risk association of *VAMP2_*rs1150 G-allele with migraine (*p* = 0.024) that was not statistically significant after Bonferroni correction (OR = 1.36; *p* = 0.068) [15]. Nevertheless, our reporter gene assay point to a functional role of this variant in gene expression. *VAMP2*_rs1150 expression quantitative trait loci (eQTLs) data suggested that the variant targets *VAMP2* expression in human brain tissues, while bioinformatics tools predicted the variant region as a target of hsa-mir-5010-3p micro-RNA [17]. Similarly, *SNAP25*_rs2327264 CT genotype showed a borderline association with migraine susceptibility (OR = 2.28; *p* = 0.003) [15]. However, no allele association was identified likely due to the small sample size, particularly the number of CC genotype subjects (*N* = 12). In our study, the reporter gene assays showed that *SNAP25*_rs2327264 C-allele influences gene expression. No eQTLs data suggested that *SNAP25*_rs2327264 targets its expression, yet this region was expected to be a target of ONECUT2 transcription factor [17]. Regarding *STX1A*_rs6951030, this variant was significantly associated with migraine (OR = 1.52; *p* = 0.006) in a previous case–control study in the Portuguese population [16] but not in a recent GWAS study [23]. In addition, it was reported an association between migraine and a haplotype that includes *STX1A*_rs6051030 [20, 21]. Nevertheless, our previous bioinformatics study predicted *STX1A*_rs6951030 (proximal enhancer) to affect the binding affinity of transcription factors from the zinc-finger protein family, namely ZNF423, and eQTLs data suggested that it disrupts *TBL2* gene expression in brain tissues [17]. *TBL2* gene encodes transducin (beta)-like 2 (TBL2), an ER transmembrane protein involved in stress-signalling and cell survival through protein synthesis regulation [24, 25]. As mentioned before, our functional assays were not able to support *STX1A*_rs6951030 impact on gene expression, possibly due to a low expression of *STX1A* and its gene regulators in the cell lines tested.

The genes studied here encode for synaptobrevin-2 (or vesicle-associated membrane protein-2; VAMP2), 25-kD synaptosome-associated protein (SNAP25), and syntaxin-1A (STX1A) proteins; all belonging to the SNARE complex that controls the docking of synaptic vesicles and potentiates presynaptic membrane fusion [18]. These proteins also interact with other elements of the exocytotic machinery and ion channels involved in the regulation of presynaptic action potentials and neurotransmitter release [18]. Several studies indicated that abnormal expression, risk genetic variants, or dysfunction of SNARE proteins are present in various neurological diseases, possibly contributing to abnormal neurotransmission and synaptic dysfunction [18]. In line with our findings, VAMP2 expression was found to be reduced in animal models or patients' brain tissues of Parkinson [26], epilepsy [27], and dementia [28]. As proposed in vascular dementia, *VAMP2*_rs1150 risk allele may have a potential role in synaptic decline and vascular alterations [28]. In these same studies, SNAP25 and STX1A expression was decreased, in opposition to our results of the *SNAP25*_rs2327264 risk allele. Nevertheless, our previous study did not find data suggesting that *SNAP25*_rs2327264 target its expression [17], so we cannot speculate further. Migraine is considered a brain state of altered excitability, therefore, changes in SNARE gene expression might alter the control of the synaptic vesicle exocytosis and consequently unbalance the release of the neuropeptides and neurotransmitters [9].

Interestingly, an in vitro study demonstrated that 4‐aminopyridine (potassium channel inhibitor) increased the rate and extent of exocytosis, and desynchronised neurotransmitter release by prolonging local calcium availability in cellular models of *VAMP2* pathogenic variants [29]. This compound has been indicated for the symptomatic treatment of multiple sclerosis, cerebellar ataxias, and Lambert–Eaton and congenital myasthenic syndrome [30]. Thus, suggesting that 4‐aminopyridine would be a highly promising treatment for patients with SNAREopathies presenting an impaired neurotransmitter release.

In conclusion, our reporter gene assays confirmed the effect of two non-coding variants in the SNARE genes *VAMP2* and *SNAP25*. In addition to the previous in silico analysis of regulatory elements, these results suggest that these non-coding variants may have implications in migraine susceptibility. Therefore, it would be interesting to understand if unbalancing the expression of genes encoding components of the synaptic vesicle machinery may disrupt the exocytosis of neuropeptides/neurotransmitters acting on the nervous system and blood vessels. Although our findings provide novel insights into the impact of non-coding variants and gene regulation of SNARE proteins, further studies are needed clarify the link between SNAREs dysregulation and migraine risk. Furthermore, our study calls attention to the importance of analysing non-coding variants, which are continuously being demonstrated to play an important role in susceptibility and complex neurological disorders.

## Data Availability

All data generated during this study are included in the manuscript.

## Abbreviations

ADHD: Attention deficit hyperactivity disorder
DMEM: Dulbecco’s modified Eagle medium
eQTLs: Expression quantitative trait loci
FBS: Fetal bovine serum
nTPM: Normalised transcript per million
PCR: Polymerase chain reaction
SD: Standard deviation
SNAP25: 25-KD synaptosome-associated protein
SNARE: Soluble N-ethylamine-sensitive factor attachment protein receptor
STX1A: Syntaxin-1A
TBL2: Transducin (beta)-like 2
VAMP2: Vesicle-associated membrane protein-2 (or synaptobrevin-2)
